# High-sensitivity in vivo contrast for ultra-low field magnetic resonance imaging using superparamagnetic iron oxide nanoparticles

**DOI:** 10.1126/sciadv.abb0998

**Published:** 2020-07-17

**Authors:** David E. J. Waddington, Thomas Boele, Richard Maschmeyer, Zdenka Kuncic, Matthew S. Rosen

**Affiliations:** 1 Institute of Medical Physics, School of Physics A28, University of Sydney, Sydney, NSW 2006, Australia.; 2 A. A. Martinos Center for Biomedical Imaging, 149 Thirteenth St., Charlestown, MA 02129, USA.; 3 ACRF Image X Institute, Faculty of Medicine and Health, University of Sydney, Sydney, NSW 2006, Australia.; 4 ARC Centre of Excellence for Engineered Quantum Systems, School of Physics, University of Sydney, Sydney, NSW 2006, Australia.; 5 The University of Sydney Nano Institute, Sydney, NSW 2006, Australia.; 6 Department of Physics, Harvard University, 17 Oxford St., Cambridge, MA 02138, USA.; 7 Harvard Medical School, 25 Shattuck St., Boston, MA 02115, USA.

## Abstract

Magnetic resonance imaging (MRI) scanners operating at ultra-low magnetic fields (ULF; <10 mT) are uniquely positioned to reduce the cost and expand the clinical accessibility of MRI. A fundamental challenge for ULF MRI is obtaining high-contrast images without compromising acquisition sensitivity to the point that scan times become clinically unacceptable. Here, we demonstrate that the high magnetization of superparamagnetic iron oxide nanoparticles (SPIONs) at ULF makes possible relaxivity- and susceptibility-based effects unachievable with conventional contrast agents (CAs). We leverage these effects to acquire high-contrast images of SPIONs in a rat model with ULF MRI using short scan times. This work overcomes a key limitation of ULF MRI by enabling in vivo imaging of biocompatible CAs. These results open a new clinical translation pathway for ULF MRI and have broader implications for disease detection with low-field portable MRI scanners.

## Teaser

Superparamagnetic nanoparticles will boost image contrast on portable MRI scanners operating at low magnetic fields.

## INTRODUCTION

Magnetic resonance imaging (MRI) has been unparalleled in its ability to noninvasively image soft tissue since it was introduced to the clinic over 30 years ago. However, decades of technical improvements have not reduced the price of an MRI scanner, which, mostly due to superconducting magnets and siting infrastructure requirements, is nominally US$1 million per tesla of magnetic field (*1*). These high costs have meant that MRI has typically been available only to relatively wealthy populations (*2*) in areas of high population density (*3*).

Recently, the clear market for a low-cost, portable MRI scanner, particularly in the field of emergency medicine, has led to the design and construction of several prototype MRI scanners that operate at low magnetic fields (<0.3 T) (*4*), where it becomes economically advantageous to generate the main *B*_0_ magnetic field with permanent magnets (*5*–*11*). Further reduction of *B*_0_ into the ultra-low field regime (ULF; *B*_0_<10 mT) (*12*) makes possible MRI scanners based on inexpensive, and lightweight, electromagnets (*13*–*16*). Using modern hardware and new acquisition techniques, this new wave of low-cost scanners can acquire far superior diagnostic information (*13*) to earlier generations of low-field MRI scanners (*17*) and could become common screening tools, particularly at remote hospitals and medical clinics (*4*).

A key challenge to the implementation of ULF MRI remains simultaneously achieving signal-to-noise ratios (SNRs) adequate for imaging and contrast-to-noise ratios (CNRs) sufficient for diagnostic differentiation between tissues within a short imaging time (*4*, *17*). The traditionally low SNR of conventional (i.e., inductively detected) ULF MRI has been improved substantially through the use of high-efficiency sequences, such as balanced steady-state free precession (bSSFP), that can accommodate rapid signal averaging (*18*). These sequences, where spins are dynamically refocused and repolarize while signal is still being acquired, have enabled diagnostically useful images of the human brain to be acquired at ULF in minutes (*13*). Unlike spin-echo–based techniques at ULF (*19*), relaxation contrast in bSSFP acquisitions is reduced due to intensity being weighted by the ratio of spin-spin and spin-lattice relaxation times (*T*_2_/*T*_1_), which converges to unity at ULF (*20*, *21*).

Here, we describe our work with biocompatible superparamagnetic iron oxide nanoparticles (SPIONs) that demonstrate high magnetizations and relaxivities in the ULF regime (*22*, *23*). We combine the high-efficiency bSSFP sequence with the unique magnetization and relaxation properties of SPIONs at ULF to enable high-sensitivity imaging of exogenous contrast agents (CAs). We exploit these magnetizations to perform sensitive, susceptibility-based SPION imaging at ULF and show how positive contrast can be generated via variations in the acquisition parameters (*24*, *25*). We also demonstrate high-contrast, preclinical imaging of SPIONs at ULF with biocompatible concentrations in a 12.5-min scan. Given the biocompatibility of SPIONs and the potential for ULF MRI to enable medical imaging in remote locations, these results could lead to new clinical applications of MRI, particularly in the field of emergency medicine.

## RESULTS

### Behavior of CAs at ULF

**Fig. 1:**
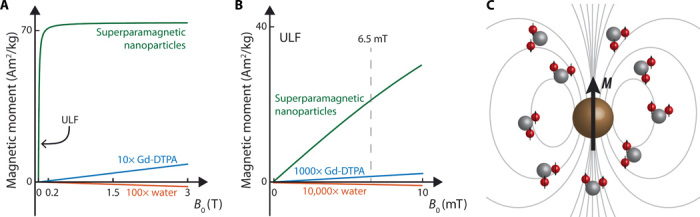
The basis of high-sensitivity SPION imaging at ultra-low magnetic fields. (**A**) Magnetization of 25-nm SPIONs (green), gadolinium CA (Gd-DTPA/Magnevist, blue), and water (red) as a function of magnetic field strength (*B*_0_). (**B**) Magnetization as a function of magnetic field strength (*B*_0_) in the ULF (<10 mT) regime for the materials shown in (A). Superparamagnetic materials, such as SPIONs, are highly magnetized even at ULF. Paramagnetic materials, such as CAs based on gadolinium, and body tissues (which typically have diamagnetic susceptibilities close to water) have absolute magnetizations that increase linearly with field strength. Curves in (A) and (B) were reproduced from data in (*32*, *53*) and reflect the magnetic moment per kilogram of compound. (**C**) Highly magnetized SPIONs (brown) interact with nearby ^1^H spins in water, shortening ^1^H relaxation times, and causing susceptibility-based shifts in Larmor frequency.

The basis of high-sensitivity CA imaging at ULF is shown in Fig. 1. Superparamagnetism, a result of the exchange interaction in iron, leads to full alignment of the magnetic moments of Fe atoms within SPIONs such that each nanoparticle behaves as one large magnetic moment of order 10,000 Bohr magnetons in magnitude. These magnetic moments are nearly fully aligned with modest external magnetic fields, giving SPION CAs magnetizations and ^1^H relaxivities that are orders of magnitude larger at ULF than paramagnetic CAs such as gadopentetic acid (Gd-DTPA), that rely on the relatively weak Zeeman interaction for magnetization alignment (see Fig. 1B). It is by leveraging these high magnetizations and relaxivities that we enable the high-sensitivity, high-contrast imaging at ULF presented here.

Commercially available magnetite (Fe_3_O_4_) SPIONs from two sources were used in this study. We focus on highly susceptible (HS) SPIONs that have 25-nm iron oxide cores functionalized with carboxylic acid (COOH) or polyethylene glycol (PEG). The behavior of these HS-SPIONs is compared to Gd-DTPA, a widely used *T*_1_ CA in clinical MRI, and ferumoxytol SPIONs [approved by the U.S. Food and Drug Administration (FDA) for anemia treatments].

The HS-SPIONs have measured magnetizations at ULF over 3000 times larger than conventional Gd-DTPA CAs (see characterization data of MRI CAs at 6.5 mT in Table 1). As the SPION magnetic moments fluctuate, they couple to nearby ^1^H nuclei (see Fig. 1C), causing a shortening of spin relaxation times. We observe that HS-SPIONs have ULF spin-lattice (*r*_1_) and spin-spin (*r*_2_) relaxivity values in excess of 300 mM ^−1^ s^−1^, nearly two orders of magnitude larger than observed for the Gd-DTPA. Relaxivity values for the HS-SPIONs are approximately 10 times that observed for ferumoxytol SPIONs, predominantly due to the higher magnetization reached in the larger iron oxide cores of the HS-SPIONs (*22*, *26*).

**Table 1:** Size, magnetization, and contrast power of different CAs at 6.5 mT. Data for 25-nm carboxylated iron oxide nanoparticles (HS-COOH), 25-nm PEGylated iron oxide nanoparticles (HS-PEG), Feraheme SPIONs (ferumoxytol, FDA-approved for iron-deficiency anemia treatments), and Magnevist (Gd-DTPA) are shown. Core sizes and hydrodynamic diameter (HD) are reproduced from (*26*, *41*) or taken from manufacturer datasheets. Magnetic moment (*M*) values at 6.5 mT were calculated per kilogram of Fe/Gd from ULF susceptibility imaging and are consistent with data available elsewhere (see Materials and Methods and note S1 for measurement details) (*22*, *26*, *32*).

**CA**	**Core (nm)**	**HD (nm)**	***M* (A·m^2^/kg Fe/Gd)**	***r*_1_ (mM^−1^ s^−1^)**	***r*_2_ (mM^−1^ s^−1^)**	***r*_2_/*r*_1_**
HS-COOH	24.2	43.4	38.5 ± 1.5	320 ± 15	365 ± 43	1.14 ± 0.13
HS-PEG	24.2	92.9	33.3 ± 1.9	261 ± 20	329 ± 45	1.26 ± 0.17
Ferumoxytol	3.3	30	4 ± 0.6	31 ± 1	31 ± 4	1.00 ± 0.13
Gd-DTPA	Gd^3+^	1.8	0.010 ± 0.002	3.4 ± 0.1	11 ± 1	3.24 ± 0.29

In tesla-strength magnetic fields, SPIONs are near exclusively used for *T*_2_-weighted imaging due to large *r*_2_/*r*_1_ ratios. The HS-SPIONs used here have *r*_2_/*r*_1_ = 208 at 3 T (see table S1 for data). We note that *r*_2_/*r*_1_ ratios (a measure of *T*_1_ contrast power) for all CAs are close to unity at 6.5 mT, which is a general property of the ULF regime (*27*). Further, we highlight that the *r*_1_ values observed at 6.5 mT for SPIONs are significantly higher than seen at clinical field strengths, which indicates the potential of SPIONs for *T*_1_-weighted imaging at ULF, especially when compared to gadolinium-based CAs (*23*, *28*).

PEGylation of HS-SPIONs has a limited impact on relaxivity despite causing a significant increase in hydrodynamic diameter (HD), indicating that long-range effects dominate ^1^H relaxation.

### In vivo imaging of SPIONs at ULF

Having characterized the high relaxivity of SPIONs at ULF, we present our first key result, the demonstration of high-sensitivity in vivo CA imaging in a 6.5-mT MRI scanner.

**Fig. 2:**
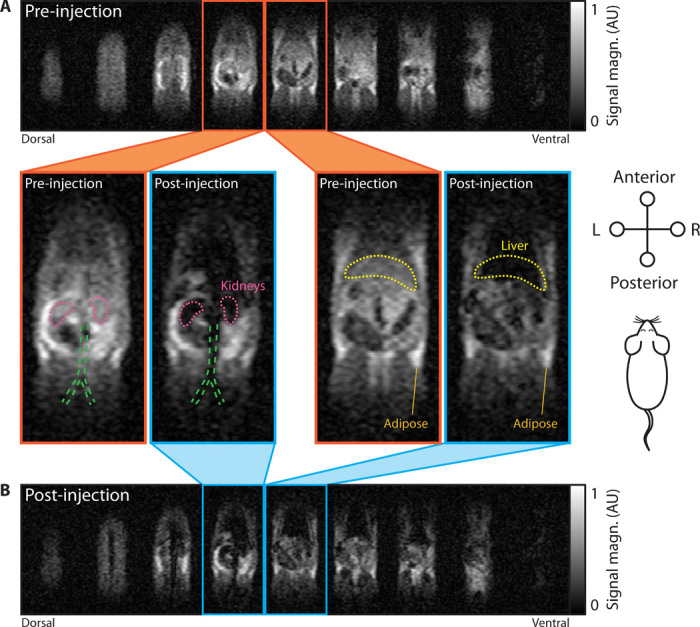
In vivo imaging at 6.5 mT of SPION biodistribution in a rat model. (**A**) MRI scan of rat anatomy before CA injection. (**B**) MRI scan of rat 30 min after a tail vein injection of HS-PEG SPIONs at 5 mg/kg. Both three-dimensional (3D) bSSFP MRI datasets were acquired in 12.5-min acquisitions with tip angle α = 90° and a 2.0 mm × 1.6 mm × 5.9 mm voxel size. The 9 signal-containing slices of 11-slice datasets are shown in (A) and (B). Attention is drawn to the two central slices from the pre-injection dataset (orange outline) and post-injection dataset (blue outline). Outlines of the kidneys (pink), liver (yellow), and the bifurcation of the aorta and inferior vena cava (green) are provided for discussion in the main text. Field of view (FOV) in each slice is 155 mm × 73 mm. AU, arbitrary units.

We begin the in vivo experiment by performing a ^1^H anatomical scan of an anesthetized Wistar rat using bSSFP MRI, as shown in Fig. 2A. At the ultra-low magnetic field strength of 6.5 mT, we measure SNRs of 24.4 ± 2.3 and 11.8 ± 1.3 in adipose and liver tissues, respectively, following this short scan (duration of 12.5 min). We note that bSSFP acquisition, which enables the sensitivity of our ULF imaging (*13*), results in images that are weighted by the ratio of *T*_2_/*T*_1_. As this ratio is nearly unity at ULF, the images shown here are essentially proton-density weighted and, as a result, have very little observable contrast between organs—an effect apparent in the pre-injection imaging in Fig. 2.

Following anatomical imaging, PEGylated HS-SPIONs were injected into the tail vein at a dose of 5 mg/kg. We observe significant negative contrast in highly perfused organs such as the kidneys and liver due to the presence of SPIONs in images acquired 30 min after injection (Fig. 2B). In particular, the MRI signal from the liver is almost entirely eliminated, giving a CNR of 6.3 when signal intensity is compared to pre-injection imaging. See fig. S1 for further data quantifying the MRI signal from organs and tissues highlighted in Fig. 2.

Having demonstrated in vivo contrast with SPIONs and bSSFP MRI at ULF, we now examine the sensitivity of the underlying contrast mechanism. For fixed *T*_R_/*T*_E_, the negative bSSFP contrast seen in Fig. 2 arises when the presence of CA causes *T*_2_ to become smaller than *T*_E_ (25 ms in this setup) (*20*). From the relaxivity data in Table 1, we calculate that this concentration threshold is reached for HS-SPIONs at 100 μM with our imaging parameters. In this in vivo experiment, the 100 μM concentration threshold is comfortably exceeded by the post-injection concentration of SPIONs in the bloodstream [calculated to be 1 mM assuming a 25-ml rat blood volume (*29*)].

The in vivo SPION dose used here equates to 90 μM Fe per kilogram of body weight, which is approximately three times lower than clinical doses used in iron replacement therapies (*30*). With our 12.5-min imaging acquisition time, this makes the techniques demonstrated here over 150 times more sensitive, in terms of minimum mass concentration threshold, than ULF nanoparticle imaging modalities based on hyperpolarization (*27*). Further, these in vivo results were obtained with voxel sizes nearly 100 times smaller and an acquisition 8 times faster than previous demonstrations of a *T*_1_-weighted approach to SPION imaging in phantoms at ULF (*23*).

We also note that SPIONs remain visible in the bloodstream until the conclusion of imaging (approximately 2 hours after injection), as evidenced by persistent negative contrast in the aorta and vena cava.

To test the potential of gadolinium as an ULF CA, we repeated the in vivo experiment at the typical maximum clinical dose of Gd-DTPA (0.2 ml/kg or 100 μM/kg). Little to no contrast resulted from the Gd-DTPA injection in our bSSFP imaging, as would be expected from the comparatively low ULF relaxivity of Gd-DTPA. We calculate that obtaining the contrast threshold of *T*_2_ < *T*_E_ in this experiment would require a bloodstream concentration of 4 mM Gd-DTPA, which is higher than can be achieved at the maximum clinical dose (see fig. S2 for in vivo Gd-DTPA imaging data and further discussion).

### Susceptibility-based imaging at ULF

The SPION imaging methods we have demonstrated thus far have been based on negative contrast induced by the high *r*_2_ relaxivity of SPIONs at ULF. Positive contrast in MRI scans is often preferred by clinicians, as negative contrast signals can be confounded by the presence of air or hemorrhage (*31*). With this in mind, we now look to the future and present a susceptibility-based positive contrast technique for ULF MRI that is directly enabled by the high magnetization of SPIONs.

**Fig. 3:**
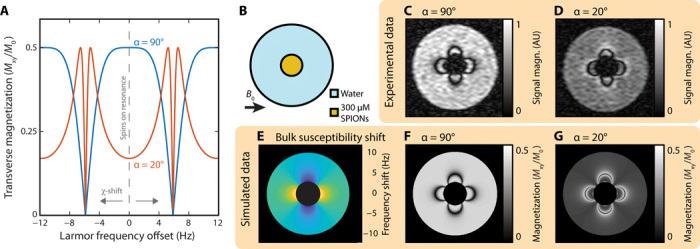
Susceptibility-based contrast at ultra-low magnetic fields. (**A**) Simulated transverse magnetization of spins during bSSFP imaging. MRI signal magnitude is proportional to the transverse magnetization (*M*_xy_) of ^1^H nuclear spins, which is shown here as a function of frequency offset (the difference between RF pulse frequency and Larmor precession frequency). Spins not in the presence of SPIONs have zero Larmor frequency offset. As SPION concentration increases, nearby spins experience a susceptibility (χ) shift in Larmor frequency. Curves for RF pulse tip angles of α = 20° (red) and α = 90° (blue) are shown. The transverse magnetization is normalized by the fully relaxed longitudinal magnetization (*M*_0_). *T*_R_ = 85 ms and *T*_1_/*T*_2_ = 1 in this model. (**B**) Phantom schematic. A small vial of HS-SPIONs at 300 μM (orange) is suspended in a larger vial of water (blue). The 6.5-mT static magnetic field of the scanner (*B*_0_) is oriented perpendicular to the cylindrical vial axis. (**C**) Standard bSSFP MRI of the phantom shown in (B), acquired at 6.5 mT with a tip angle α = 90°. (**D**) Small tip angle bSSFP MRI of the same phantom, acquired with α = 20°. (**E**) Analytical calculation of the ^1^H Larmor frequency shift expected in the water region of the phantom in (A) due to bulk magnetization of the adjacent SPION vial. (**F**) Signal magnitude predicted in a bSSFP MRI of ^1^H spins shifted by frequencies depicted in (E) when α = 90°. (**G**) Signal magnitude predicted in a bSSFP MRI of ^1^H spins shifted by frequencies depicted in (E) when α = 20°. Simulation data for the SPION vial are blacked out in (E) and (F) as well as (G) as a key assumption of the bSSFP trajectory model, that *T*_R_ ≪ *T*_2_, does not hold in this region of the images. FOV in all images is 49 mm. The α = 90° and α = 20° images were acquired with 6.2- and 12.4-min scans, respectively.

The basis of positive contrast demonstrated here is the shift in Larmor resonance frequency of ^1^H spins induced by the local magnetic field of magnetized SPIONs (as illustrated in Fig. 1). In a bSSFP acquisition, the MRI signal magnitude is directly proportional to the steady-state transverse magnetization of ^1^H spins, which, in turn, depends strongly on the radiofrequency (RF) pulse tip angle (α) used as well as the offset, *f*_offset_, between Larmor frequency and RF pulse frequency (see Fig. 3A) (*24*).

Typically, bSSFP scans are acquired at, or close to, α = 90° so that imaged ^1^H spins lie on the plateau around *f*_offset_ = 0 Hz and undesired “banding” artifacts due to shifts in Larmor resonance frequency, typically caused by magnetic field inhomogeneities, are suppressed (*20*). However, the high absolute magnetic field homogeneity of low-field magnets makes the ULF regime inherently robust to these banding artifacts (*13*), allowing us to exploit smaller bSSFP tip angles (such as α = 20°) that selectively boost the MRI signal from ^1^H spins as they are shifted off resonance by the bulk susceptibility effects of SPIONs (*25*).

To demonstrate the positive contrast boost that results from off-resonance spins, we image a phantom containing HS-SPIONs suspended in water (see Fig. 3B). When α = 90° is used (Fig. 3C), water surrounding the vial of SPIONs displays dark bands at ^1^H frequency shifts of ±1/2*T*_R_ (±6 Hz) overlaid on a uniform background signal intensity. Imaging with a low α of 20° (Fig. 3D) reveals the vial with positive contrast as signal boosting occurs adjacent to these same dark bands.

By fitting imaging data to the bSSFP signal model (Fig. 3A) and approximating the vial of SPIONs as a long cylinder, we calculate the SPION magnetization (values shown in Table 1) and the spatial, susceptibility-based shift in ^1^H frequency (see Fig. 3E). Details of the magnetization fitting procedure, which is adapted from (*32*), are given in note S1 in addition to further experimental data. We note that the images simulated from the model for 90° and 20° bSSFP acquisitions (Fig. 3, F and G) show agreement with experimental images (Fig. 3, C and D).

**Fig. 4:**
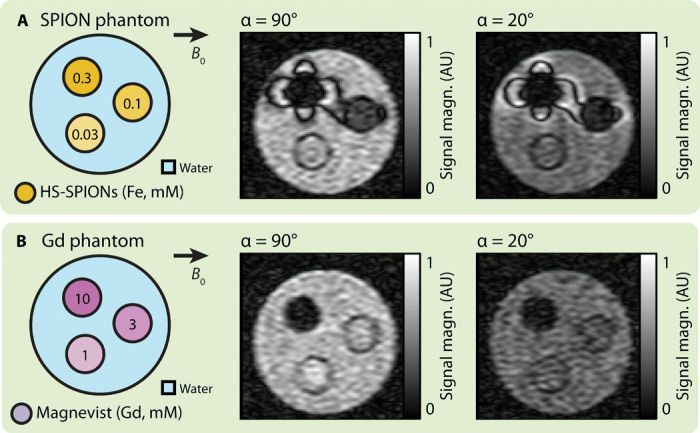
Sensitivity of CA imaging at ULF. (**A**) Phantom schematic (left) shows vials of HS-COOH SPIONs at various concentrations (orange) in a larger water vial (blue). bSSFP MRI scans of the phantom with α = 90° (middle) and α = 20° (right) are also shown. (**B**) Phantom schematic (left) shows vials of Magnevist at various concentrations (purple) in a larger water vial (blue). bSSFP MRI scans of the phantom with α = 90° (middle) and α = 20° (right) are also shown. FOV in all images is 49 mm. The α = 20° SPION image was acquired with a 12.4-min scan. All other images were acquired with 6.2-min scans. AU, arbitrary units.

Having shown susceptibility-based positive contrast at ULF, we now turn to quantify the concentration sensitivity of the technique by imaging the HS-SPION phantom shown in Fig. 4A. We observe that negative contrast within the SPION solution, due to *T*_2_-shortening effects, begins to manifest at the same 0.1 mM concentration threshold at which positive contrast in adjacent structures becomes visible in our α = 20° scan. The concentration threshold for imaging ferumoxytol SPIONs with negative and positive contrast (∼1 mM) is higher than for HS-SPIONs, as would be expected from their lower relaxivity and magnetization at ULF (supporting data in fig. S3).

Repeating the two tip angle imaging experiments with a phantom containing Gd-DTPA (Fig. 4B), we observe that negative, relaxivity-based contrast is present at a relatively high concentration of 10 mM. However, no positive contrast is observed from Gd-DTPA in the α = 20° scan, as the magnetization of the paramagnetic compound is too low. We note that positive contrast can be observed in an α = 20° scan of Gd-DTPA by increasing the concentration to a clinically unfeasible 500 mM (see fig. S4 for data).

### In vivo susceptibility-based contrast at ULF

Mindful that phantoms typically enhance MRI contrast, we now demonstrate positive-contrast imaging with SPIONs at ULF in our healthy rat model.

**Fig. 5:**
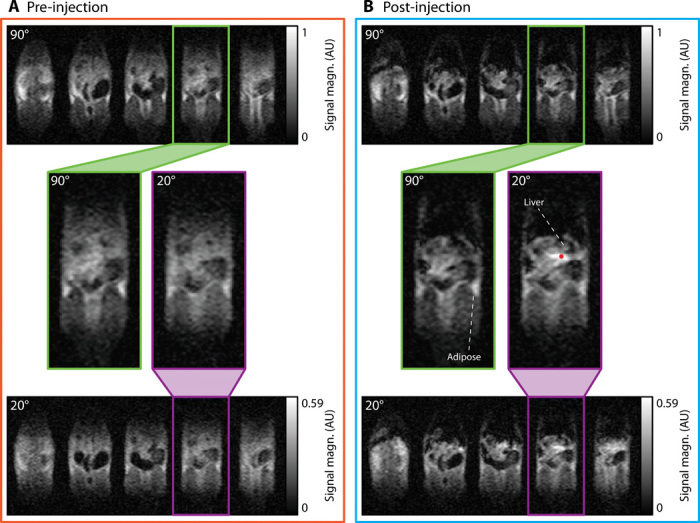
Switchable susceptibility-based SPION contrast. (**A**) Images of rat anatomy before CA injection acquired with bSSFP tip angles of α = 90° (top) and α = 20° (bottom). (**B**) MRI scans taken 30 min after a tail vein injection of HS-COOH SPIONs at 5 mg/kg with α = 90° (top) and α = 20° (bottom). The 5 central slices of 11-slice datasets are shown for each acquisition. Expanded images of individual slices from α = 90° (green outline) and α = 20° (purple outline) datasets are shown. FOV in each slice is 155 mm × 73 mm. The red dot below the liver indicates a region of “boosted” signal for further discussion in the text. The 3D datasets with α = 90° were acquired with 12.5-min scans. The 3D datasets with α = 20° were acquired with 25-min scans. AU, arbitrary units.

Immediately after anesthetization, pre-injection anatomical scans were acquired with α = 90° and α = 20° (shown in Fig. 5A). Comparing pre-injection scans, we observe that time-weighted SNR of adipose tissue in the 20° scan is a factor of 2.9 lower than in the 90° scan, which is consistent with the reduction in transverse magnetization for on-resonance spins predicted from Fig. 3A. However, contrast between tissues in 90° and 20° scans is qualitatively identical, as all spins are on-resonance and transverse magnetization is suppressed uniformly.

Following anatomy scans, HS-SPIONs functionalized with COOH were administered at 5 mg/kg and 30 min allowed biodistribution of SPIONs. In post-injection images (Fig. 5B), clear differences between 90° and 20° acquisitions are immediately apparent. While the 90° images show negative contrast in the liver and kidneys, as observed in Fig. 2, the 20° images show positive contrast around regions of SPION uptake due to signal boosting of off-resonance spins.

We see, for example, that SNR increases by 1.7 in tissue posterior to the liver when compared to the 20° pre-injection scan (Fig. 5A). Tissue anterior to the liver will experience a post-injection resonance shift, as it also lies along the dipole axis of magnetized SPIONs. However, this anterior region appears dark in the post-injection 20° scan, likely due to significant concentrations of SPIONs causing *T*_2_ shortening in this highly vascularized tissue. We note that MRI signal from, and around, tissues that have minimal SPION uptake, such as adipose tissue (*33*), is again suppressed when imaged with α = 20°.

Unlike the PEGylated SPIONs used for imaging in Fig. 2, the HS-COOH SPIONs used for contrast in Fig. 5 are not visible in the vena cava at the conclusion of imaging. This difference is likely due to the rapid removal from the bloodstream of nanoparticles with negative zeta potential, such as carboxylated SPIONs, by the immune system (*34*).

## DISCUSSION

SPIONs are routinely prescribed clinically at concentrations above those used in this in vivo study (*35*). Hence, the results presented here illustrate the clinical potential of SPIONs as ULF CAs that give sensitive contrast with time-efficient imaging techniques. Although our in vivo SPION imaging experiments were restricted to HS-SPIONs for their high magnetization at 6.5 mT, all techniques demonstrated are immediately applicable to ferumoxytol SPIONs. Given the common off-label use of ferumoxytol as a high-field clinical MRI CA for imaging a diverse range of anatomical sites (*36*), we foresee few barriers to clinical translation of our bSSFP-based ULF SPION imaging (*37*, *38*), especially with the imminent commercial availability of low-field MRI scanners (e.g., as manufactured by Hyperfine Research Inc. and Promaxo Inc.) and increasing concerns over the neuroaccumulation of gadolinium CAs (*39*).

Because of the nonlinearity of superparamagnetism, the HS-SPIONs used here reach 30% of their field-saturated magnetization at 6.5 mT (*22*), enabling the use of susceptibility-based contrast techniques at ULF. We note that due to the negligible magnetizations induced in human tissues at low magnetic field, susceptibility-weighted imaging has never previously been demonstrated in ULF MRI (*40*). Further, we expect that the accuracy of SPION-based diagnosis will be aided by the suppression of background susceptibility artifacts at ULF—a challenge that has hindered the widespread use of SPIONs at high fields (*41*).

Given that the ULF MRI platform used in this study has previously been used for three-dimensional (3D) imaging of the human brain in 6 min, despite the 460-fold reduction in field strength from 3 T (*13*), we anticipate that human scans using SPIONs will be possible over similar time frames. In addition, opportunities exist to increase SNR and CNR in future implementations. Beyond the tip angle–based approach we have demonstrated in vivo, variations in *T*_R_ and transmit frequency may also be used to selectively probe regions of SPIONs with additional positive contrast at lower concentrations (see figs. S5 and S6) (*25*). Calculating difference images via postprocessing techniques may also prove useful in identifying regions of significant SPION uptake (shown in fig. S7).

The continued development of such positive contrast approaches will be vital to the unambiguous identification of SPIONs in ULF images. The contrast techniques shown here at 6.5 mT can also be extended to low-field MRI scanners based on permanent magnets. Iron oxide nanoparticle size may also be customized to maximize magnetization and ^1^H relaxivity at the desired field strength. Any modification to nanoparticle composition for use of SPIONs as blood pool CAs, of use in clinical applications including brain MRI (*42*), must incorporate steps to limit sequestration of nanoparticles by the liver (*43*). Typical steps to limit liver sequestration include the use of SPIONs 100 nm or smaller in size and functionalizing the nanoparticle surface with a biocompatible polymer such as PEG (*44*).

Further approaches to increasing SNR and CNR at ULF include the use of sensitive superconducting quantum interference devices (SQUIDs) (*45*) or hyperpolarization techniques that can boost nuclear spin polarizations by orders of magnitude (*46*, *47*). Both of these approaches face substantial challenges; SQUID-based MRI typically requires a time-consuming prepolarization step (*19*), and the toxicity of hyperpolarization agents must be considered (*27*, *48*, *49*).

The contrast mechanisms presented here will likely also prove useful for the integration of MRI with new theranostic modalities, including magnetic particle imaging (*50*) and superparamagnetic relaxometry (*51*) that use the unique properties of SPIONs at ULF. These new modalities, which may prove vital in the future for early detection and diagnosis of diseases such as cancer, require a complementary system for anatomical imaging such as a computed tomography (CT) or ULF MRI scanner due to incompatibilities with the high magnetic fields of clinical MRI scanners.

In conclusion, these results represent the most sensitive ULF contrast technique that we are aware of and will be immediately applicable to other implementations of portable and low-field MRI. Given their unique low-field properties, we expect tailored SPIONs to become the CA of choice for ULF MRI.

## MATERIALS AND METHODS

### ULF MRI system

All measurements were made in an open-access ULF MRI scanner that has been described previously (*13*). This scanner consists of a biplanar electromagnet operating at a 6.5-mT magnetic field and biplanar magnetic gradient coils. Gradient control, RF pulse generation, and data acquisition were performed with a Redstone NMR Spectrometer (Tecmag, Houston, TX, USA). A 500-W pulsed power amplifier (BT00500-AlphaS, TOMCO Technologies, Stepney, SA, Australia) was used for ^1^H pulses at 276 kHz. The homebuilt gradient set and Techron 7782 gradient amplifiers (Elkhart, IN, USA) were used to generate gradient fields as high as 1 mT m^−1^.

### Contrast agents

HS-SPIONs with carboxylic acid (HS-COOH) and PEG (HS-PEG) outer coatings were obtained from Imagion Biosystems (PrecisionMRX product line). These nanoparticles consist of a 25-nm iron oxide core encapsulated in polymer (40 nm outer diameter) (*22*). FDA-approved for iron-deficiency anemia treatment and commercially available ferumoxytol, SPIONs (Feraheme) were obtained clinically for comparison. The imaging performance of SPIONs at ULF was also compared to that of Gd-DTPA (Magnevist) from Bayer Schering Pharma.

CAs were diluted in deionized water for spectroscopic measurements and phantom imaging. CAs were diluted in 0.9% saline for in vivo injection.

### Spectroscopic relaxivity measurements

The *T*_1_ and *T*_2_ relaxation times of ^1^H nuclei were measured in aqueous solutions of CAs at a range of concentrations. *T*_1_ and *T*_2_ relaxation times were measured using conventional saturation recovery and Hahn echo sequences, respectively. The longitudinal relaxivity (*r*_1_) and transverse relaxivity (*r*_2_) of CAs were extracted by fitting the relaxation times of CAs to the concentration-dependent relaxivity equation. See note S2 for further details. All 6.5-mT spectroscopic measurements were performed in a 30-mm-diameter NMR coil tuned and matched at 276 kHz.

### ULF imaging

Imaging was performed at 6.5 mT (^1^H = 276 kHz) using a homebuilt probe designed for rat body imaging (shown in fig. S8). This probe consists of a two-layer solenoid wound on a hollow polycarbonate former that is 90 mm in length and 64 mm in outer diameter (60 mm in inner diameter). The solenoidal coil was wound using 5/39/42-AWG20 Litz wire with fluorinated ethylene propylene insulation (New England Wire Technologies, Lisbon, NH, USA), chosen for its low resistivity at 276 kHz. The coil was tuned to 276 kHz using an external resonator board (parallel tune—series match) and resistively broadened to an *s*_21_ bandwidth of 4.7 kHz. This bandwidth allows a maximum 11-cm field of view (FOV) when imaging hydrogen nuclei with our 1 mT/m gradient set (*49*).

In this study, we define directions with respect to anatomical planes and not the magnetic field (*B*_0_). The orientation of rat imaging in our biplanar ULF magnet means that axial and coronal directions are perpendicular to *B*_0_, while the sagittal direction is parallel to *B*_0_.

All data were acquired with a high-efficiency 3D bSSFP sequence that has been described previously (*13*). All data were pseudorandomly undersampled by 50% to accelerate acquisition (*18*). Phantom images were acquired with axial slices and the following parameters: matrix size (readout × phase encode 1 × phase encode 2) = 256 × 45 × 12, resolution = 1.1 mm × 1.1 mm × 8.9 mm, α = 90°, *T*_R_/*T*_E_ = 85/42 ms, and number of averages (NA) = 16 for a 6.2-min acquisition. Animal images were acquired with coronal slices and the following parameters: matrix size = 128 × 45 × 11, resolution = 2.0 mm × 1.6 mm × 5.9 mm, α = 90°, *T*_R_/*T*_E_ = 50/25 ms, and NA = 60 for a 12.5-min acquisition. Axial and coronal images were zero-filled to in-plane resolutions of 0.5 mm × 0.6 mm and 1.0 mm × 0.8 mm, respectively, for display. Some images were acquired with the parameters listed above but α = 20°. NA was doubled for some of these α = 20° acquisitions, increasing the acquisition time by a factor of 2. Acquisition times for each image are specified in figure captions.

We calculate the SNR as the mean magnitude of the MRI signal in a region of interest (ROI) divided by the root mean square value of the MRI signal in an empty background region. We define the CNR as the difference between mean MRI signal in two ROIs divided by the root mean square value of the MRI signal in an empty background region. We note that CNR calculations quantifying the change in signal intensity between pre- and post-injection images use the same acquisition parameters for both scans so that the noise floor is of equivalent magnitude.

### Magnetization simulations

The magnetizations of SPION and Gd-based CAs were calculated at 6.5 mT by comparing experimental imaging data to analytical solutions for the field patterns associated with vials of CA in an external magnetic field (*32*). In this comparison, the theoretical susceptibility shift in *B*_0_ magnetic field and ^1^H Larmor frequency is first calculated for the region surrounding an infinitely long cylinder of paramagnetic CA (*52*). The ^1^H resonance frequency shift is then used to simulate image patterns expected in water surrounding the CA agent vial by calculation of the predicted bSSFP steady-state signal amplitude for known values of *T*_1_, *T*_2_, *T*_R_, and α (*20*). These simulations generate regions of null signal, known as banding artifacts, that occur at susceptibility-shifted frequency intervals of 1/*T*_R_. The CA magnetization was scaled to the image data, and the sum of squares for the difference between pixel intensities was minimized to fit the banding artifacts in simulations to those in experimental data. See note S1 for further details of susceptibility shift calculations.

### Animal preparation and study

All experiments were performed in accordance with our institutional animal care and use committee guidelines. Under anesthesia (2% isoflurane), a tail vein catheter was placed into a 300 ± 25 g male Wistar rat. After catheterization, the animal was transferred to the custom rat body imaging coil. An integrated sliding bite bar with anesthesia nose cone was used to maintain general anesthesia during experiments and to align the rat with the isocenter of the scanner. Oxygen saturation as well as cardiac and respiratory rates were continuously monitored during experiments with a Model 1025T Monitoring and Gating System (Small Animal Instruments, Stony Brook, NY, USA).

After transfer to the MRI scanner, the rat was immediately imaged with a bSSFP sequence (once with α = 90° and once with α = 20°). A 1-ml bolus of CA diluted in saline was then administered via the tail vein over 30 s and followed by 1 ml of 0.9% saline. For SPION-based CAs, the total dose of Fe was 1.5 mg, which corresponds to 5 mg/kg. For Gd-based contrast, the total dose of Gd was 8 mg, which corresponds to 60 μl (or 0.2 ml/kg) of Magnevist. The animal was then repeatedly imaged with bSSFP MRI (α = 90° or 20°) until the end of the experiment. Four rats, judged to be the minimum number sufficient to demonstrate the ULF contrast techniques, were used in the in vivo study (HS-PEG, two rats; HS-COOH, one rat; Gd-DTPA, one rat).

At the conclusion of the experiment, the animals were euthanized under deep anesthesia by isoflurane overdose. Death was confirmed by cervical dislocation. Total time from onset of anesthesia to euthanization was limited to 4 hours.

## Supplementary Material

abb0998_SM.pdf

## SUPPLEMENTARY MATERIALS

Supplementary material for this article is available at http://advances.sciencemag.org/cgi/content/full/6/29/eabb0998/DC1

## Appendix

View/request a protocol for this paper from *Bio-protocol*.
