# Supplementary material for: High-sensitivity in vivo contrast for ultra-low field magnetic resonance imaging using superparamagnetic iron oxide nanoparticles
Source: Sci Adv. 2020 Jul 17;6(29):eabb0998. doi: 10.1126/sciadv.abb0998 (PMC7367688; doi:10.1126/sciadv.abb0998)
Supplement: abb0998_SM.pdf [file abb0998_SM.pdf]

Science Advances
AAAS

advances.sciencemag.org/cgi/content/full/6/29/eabb0998/DC1

Supplementary Materials for

High-sensitivity in vivo contrast for ultra-low field magnetic resonance imaging using superparamagnetic iron oxide nanoparticles

David E. J. Waddington*, Thomas Boele, Richard Maschmeyer, Zdenka Kuncic, Matthew S. Rosen

*Corresponding author. Email: david.waddington@sydney.edu.au

Published 17 July 2020, *Sci. Adv.* **6**, eabb0998 (2020)
DOI: 10.1126/sciadv.abb0998

This PDF file includes:

Notes S1 and S2
Table S1
Figs. S1 to S8

Supplementary Note 1: Magnetization Calculations Based on Susceptibility Effects

To determine the magnetization (M) of contrast agents (CAs) at 6.5 mT, bSSFP MRI images with banding artifacts caused by CA magnetization were acquired. The measured data were fit with simulated images calculated from equations for magnetization of CA vials and the bSSFP MRI signal equation. Examples of acquired and simulated images are shown in Figure 3.

To simulate image artifacts we used the model presented in reference 27, which is an analytical calculation of the magnetic field produced by an infinitely long vial of CA placed transverse to a static, x-directed, magnetic field (B_0). Defining B_0 in the x-direction is unconventional in MRI but appropriate in these calculations as it means the z-direction is parallel to the axis of the CA vial and conventional cylindrical coordinates may easily be used. For the water region of the phantom shown in Fig. 3b, the change in magnetic field (ΔB_x) induced by magnetization of the CA is given by:

$$\Delta B_x = \mu_0 \left(1 + \chi_3\right) \frac{D_3}{r^2} \cos 2\varphi \tag{1}$$

where D_3 is a constant given by equation (2) for gadolinium based contrast agents and equation (3) for iron-oxide based contrast agents.

$$D_3 = \frac{H_0 R_2^2 \left(R_2^2 \left(2 + \chi_1 + \chi_2\right)\left(\chi_2 - \chi_3\right) + R_1^2 \left(\left(\chi_1 - \chi_2\right)\left(2 + \chi_2 + \chi_3\right)\right)\right)}{R_1^2 \left(\chi_1 - \chi_2\right)\left(\chi_2 - \chi_3\right) + R_2^2 \left(2 + \chi_1 + \chi_2\right)\left(2 + \chi_2 + \chi_3\right)} \tag{2}$$

$$D_3 = -\frac{R_2^2 \left(2 M_s R_1^2 \left(1 + \chi_2\right) - H_0 \left(-R_2^2 \left(2 + \chi_2\right)\left(\chi_2 - \chi_3\right) + R_1^2 \chi_2 \left(2 + \chi_2 + \chi_3\right)\right)\right)}{R_1^2 \chi_2 \left(\chi_2 - \chi_3\right) - R_2^2 \left(2 + \chi_2\right)\left(2 + \chi_2 + \chi_3\right)} \tag{3}$$

In these equations, χ_1 is the magnetic susceptibility of the region containing contrast agent ($0 < r < R_1$), χ_2 is the susceptibility of the glass vial ($R_1 < r < R_2$) and χ_3 the susceptibility of water ($r > R_2$). The variables r and φ are cylindrical coordinates defined relative to the center of the phantom and the B_0 field direction. R_1 and R_2 are the inner and outer radii of the CA vial. H_0 is the applied magnetic field strength and M_s is the CA magnetization. μ_0 is the permeability of free space.

From Equation 1, the shift in Larmor resonance frequency can be calculated ($\Delta f_{\mathrm{L}} = \gamma_{\mathrm{H}} \Delta B_x$). This frequency shift was used in conjunction with the repetition time (T_{R}) and tip angle (α) to

produce an analytical bSSFP image using the bSSFP signal equation [20], assuming $T_1/T_2 = 1$ which is true at 6.5 mT [17]:

$$M_{\mathrm{xy}}(\Delta f, \alpha) = M_{\mathrm{o}} \frac{\sin\alpha \cdot \sqrt{2 + 2\cos(2\pi \cdot \Delta f \cdot T_{\mathrm{R}})}}{2\,(T_1/T_2)\,(1 - \cos\alpha) + [1 + \cos(2\pi \cdot \Delta f \cdot T_{\mathrm{R}})](1 + \cos\alpha)} \quad (4)$$

All values except the magnetization of the CA ($M = M_s \frac{H}{|H|}$ for SPIONs and $M = \chi_1 H$ for Gd) are known for phantom images. Experimental images were fit by analytical images via interpolation of simulated data onto an equivalent resolution grid for different values of M_s. The magnetization value that gave the highest 2-D correlation coefficient (corr2 function in MATLAB) between water regions in experimental and simulated data was selected as the best fit. Magnetization error bars were calculated as the change in magnetization required for a 10% drop in 2-D correlation coefficient.

Supplementary Note 2: Relaxivity

The longitudinal relaxivity (r_1) and the transverse relaxivity (r_2) of contrast agents were determined by fitting T_1 (inversion recovery) and T_2 (Hahn echo) measurements, respectively, to the concentration dependent relaxivity equation:

$$1/T_{1,2} = 1/{T^0}_{1,2} + R_{1,2}[\mathrm{CA}] \quad (5)$$

Where $T_{1,2}$ is the ^{1}H relaxation time of the solution with CA, ${T^0}_{1,2}$ is the ^{1}H relaxation time in the absence of contrast agent, $r_{1,2}$ is the relaxivity coefficient, and [CA] is the concentration of contrast agent.

	Magnetic Field Strength (T)	
	3	7
r_1 ($mM^{-1}s^{-1}$)	0.86±0.13	0.44±0.13
r_2 ($mM^{-1}s^{-1}$)	179±12	585±9

Table S1. **SPION relaxivity at clinical field strengths.** This table shows the relaxivity values of carboxylated, highly susceptible (HS) SPIONs measured in preclinical 3 T and 7 T MR Solutions MRI scanners. T_1 and T_2 values were obtained by fitting phantom imaging data acquired with a fast spin echo (FSE) sequence to standard magnetization recovery (T_1) and magnetization decay (T_2) models for various T_R/T_E.

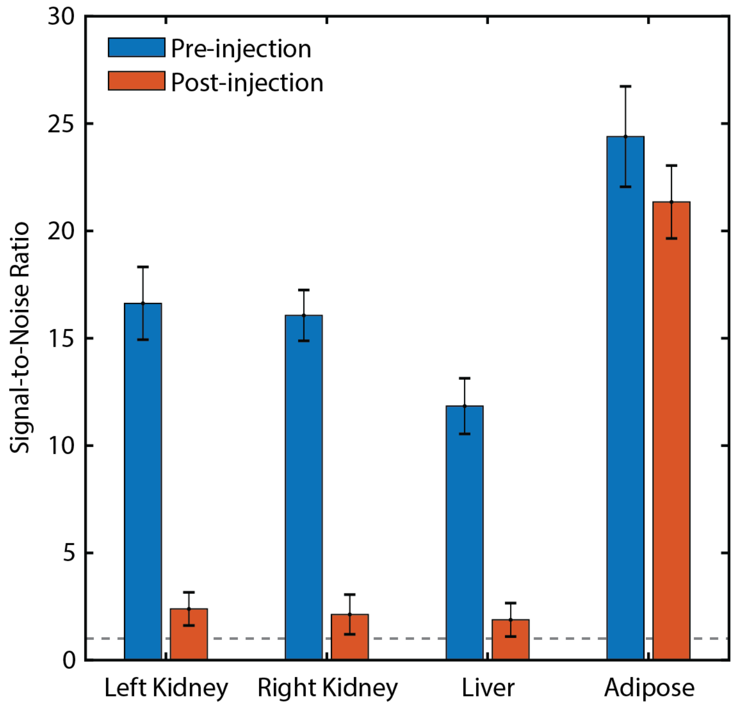

Figure S1. **Signal-to-noise ratio of tissues before and after SPION injection.** Columns indicate the mean signal-to-noise ratio (SNR) of the pre-injection (blue) and post-injection (red) MRI signal from organs/tissues in Figure 2 of the main manuscript. The black bars represent the standard deviation of the SNR within an organ/tissue. Signal values were calculated by segmenting the organs/tissues into regions of interest (ROIs) in MATLAB. Mean SNR was calculated as the mean magnitude of the MRI signal in an ROI divided by the root mean square value of the MRI signal in an empty background region (the image noise floor). The horizontal gray dotted line (SNR = 1) indicates the image noise floor.

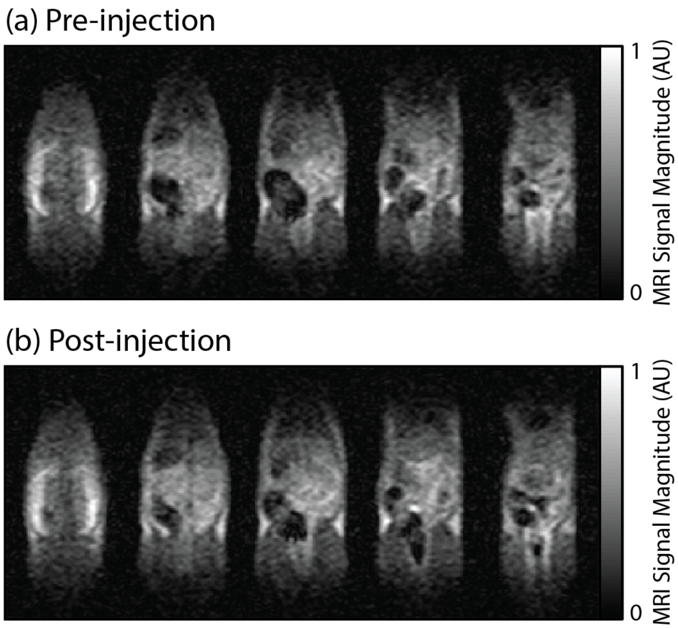

Figure S2. **Ultra-low field imaging of Gd-DTPA in a healthy rat model.** The *in vivo* biodistribution study was repeated with Gd at the typical maximum clinical dose of 0.2 mL/kg (corresponding to a Gd dose of 0.1 mM/kg body weight. **(a)** MRI scan of rat anatomy before contrast agent injection. **(b)** MRI scan of rat one hour after a tail-vein injection of Magnevist (Gd-DTPA) at 0.2 mL/kg. Both 3D bSSFP MRI datasets were acquired in 12.5 minute acquisitions with $\alpha = 90°$, $T_R/T_E = 50/25$ ms and a $2.0 \times 1.6 \times 5.9$ mm^3 voxel size. The 5 central slices of 11-slice datasets are shown in (a) and (b). Field of view in each slice is 155 mm $\times$ 73 mm. Little to no difference in MRI signal is seen as a result of Gd-DTPA injection, likely due to the relatively low r_2 relaxivity of Gd-DTPA at 6.5 mT. We estimate that contrast would arise above 3.6 mM Gd-DTPA concentration (when T_2 becomes smaller than T_E) based on relaxivity data in Table I) but Gd-DTPA only reaches an *in vivo* blood concentration of 1.2 mM (assuming all contrast agent is initially distributed in the $\sim$25 mL blood volume of the rat [28]).

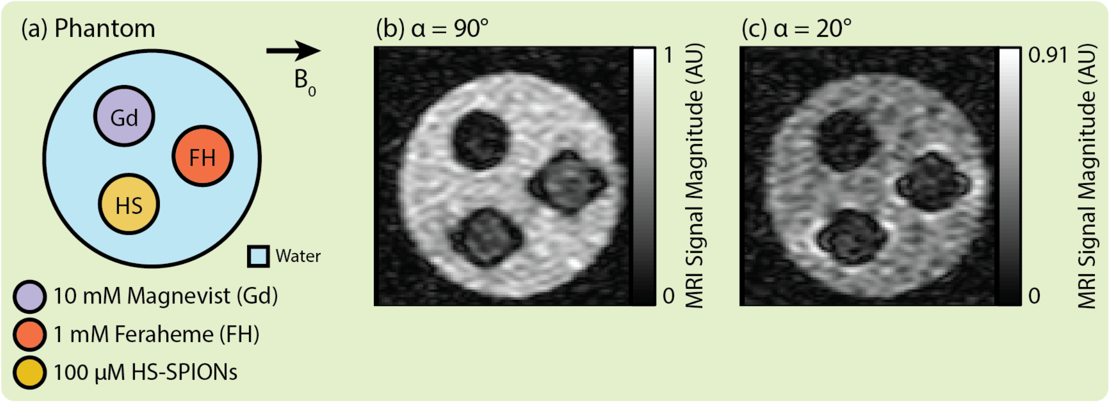

Figure S3. **ULF MRI with comparable negative contrast from gadolinium and SPIONs at different concentrations.** **(a)** Phantom schematic. Small vials of 10 mM Magnevist (Gd, purple), 1 mM Feraheme (FH, orange) and 100 µM highly susceptible (HS) SPIONs are suspended in a larger vial of water (blue). The static 6.5 mT magnetic field (B_0) is oriented perpendicular to the cylindrical vial axis. Concentrations were selected to for similar T_1 and T_2 based on the ULF relaxivity values in Table 1. **(b)** Standard bSSFP MRI of the phantom shown in panel (a), acquired with $\alpha = 90°$. **(c)** Small tip angle bSSFP MRI of the same phantom, acquired with $\alpha = 20°$. Field of view in all images is 49 mm. Images were acquired with separate 6.2 min scans.

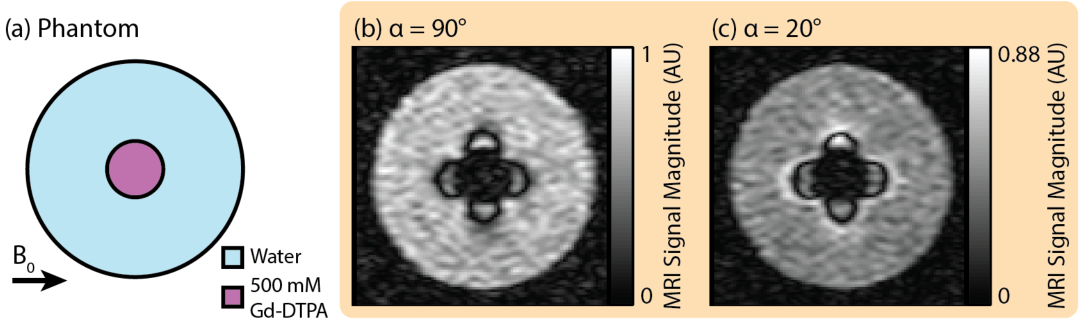

Figure S4. **Imaging Gd-DTPA at ULF at high concentrations.** **(a)** Phantom schematic. A small vial of 100% Magnevist (500 mM Gd-DTPA, purple) is suspended in a larger vial of water (blue). The static 6.5 mT magnetic field (B_0) is oriented perpendicular to the cylindrical vial axis. **(b)** Standard bSSFP MRI of the phantom shown in panel (a), acquired with $\alpha = 90°$. **(c)** Small tip angle bSSFP MRI of the same phantom, acquired with $\alpha = 20°$. Field of view in all images is 49 mm. The $\alpha = 90°$and $\alpha = 20°$images were acquired with 6.2 min and 12.4 min scans, respectively.

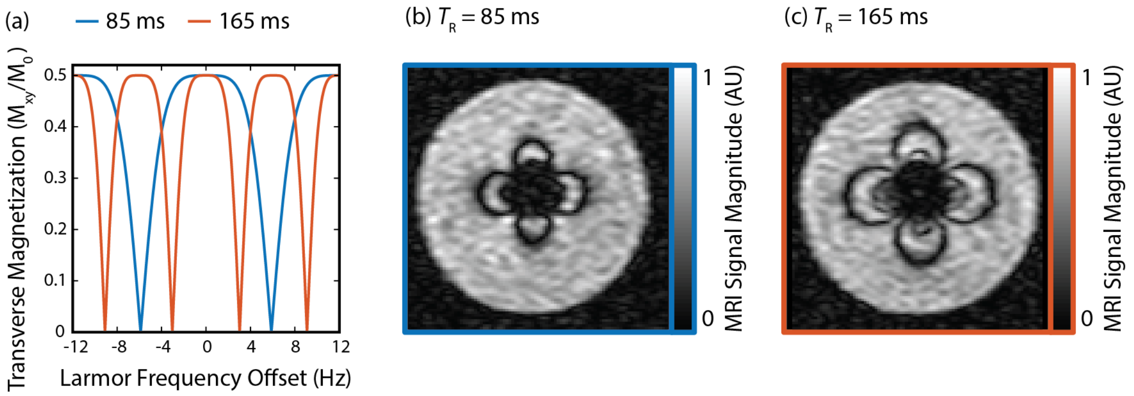

Figure S5. **Impact of T_{R} on transverse magnetization and susceptibility-based contrast at ULF.** **(a)** The transverse magnetization (M_{xy}) of ^{1}H nuclear spins as a function of frequency offset for $T_{\mathrm{R}} = 85$ ms (blue) and $T_{\mathrm{R}} = 165$ ms (red). Increasing T_{R} leads to smaller magnetization plateaus and significant changes in MRI signal for ^{1}H spins with smaller frequency offsets. $\alpha = 90°$ and $T_1/T_2 = 1$ in this model [20]. **(b)** bSSFP imaging at 6.5 mT with $T_{\mathrm{R}} = 85$ ms and $\alpha = 90°$ of a vial of 300 µm highly susceptible (HS) SPIONs centered in a larger vial of water. **(c)** bSSFP imaging at 6.5 mT with $T_{\mathrm{R}} = 165$ ms and $\alpha = 90°$ of a vial of 300 µm HS-SPIONs centered in a larger vial of water. This image has more extensive contrast due to the presence of SPIONs than is visible in (b), with ^{1}H spins in the water vial showing signal changes at smaller frequency offsets. This improved contrast comes at the cost of an approximately 2× increase in acquisition time due to the longer T_{R}. Field of view in all images is 49 mm. Images in (a) and (b) were acquired with 6.2 min and 12.0 min scans, respectively.

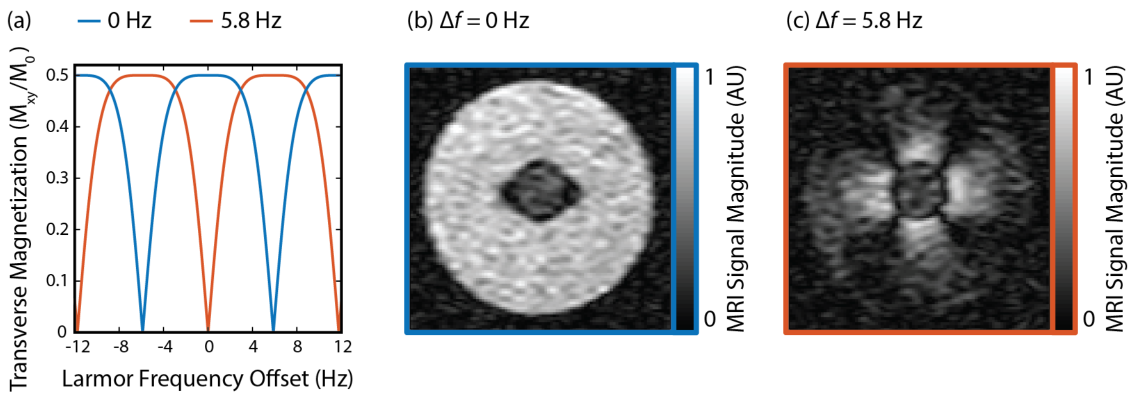

Figure S6. **Impact of pulse frequency offset on transverse magnetization and susceptibility-based contrast at ULF. (a)** The transverse magnetization (M_{xy}) of ^{1}H nuclear spins as a function of frequency offset for $T_{\mathrm{R}} = 85$ ms when the RF drive pulses are at the Larmor frequency (0 Hz, blue) and shifted from the Larmor frequency by $1/2T_{\mathrm{R}}$ (5.8 Hz, red). Shifting the RF drive frequency by $1/2T_{\mathrm{R}}$ quenches the MRI signal from on-resonance spins whilst maintaining the MRI signal from spins off resonance. $\alpha = 90°$ and $T_1/T_2 = 1$ in the model curves plotted [20]. **(b)** bSSFP imaging at 6.5 mT with no RF drive offset ($\Delta f = 0$ Hz), $T_{\mathrm{R}} = 85$ ms and $\alpha = 90°$ of a vial of 100 μm highly suceptible (HS) SPIONs centered in a larger vial of water. **(c)** bSSFP imaging at 6.5 mT with an RF drive offset $1/2T_{\mathrm{R}}$ ($\Delta f = 5.8$ Hz), $T_{\mathrm{R}} = 85$ ms and $\alpha = 90°$ of a vial of 100 μm HS-SPIONs centered in a larger vial of water. Signal is only visible from ^{1}H spins shifted off-resonance due to the presence of SPIONs. Field of view in all images is 49 mm. Images were acquired with separate 6.2 min scans.

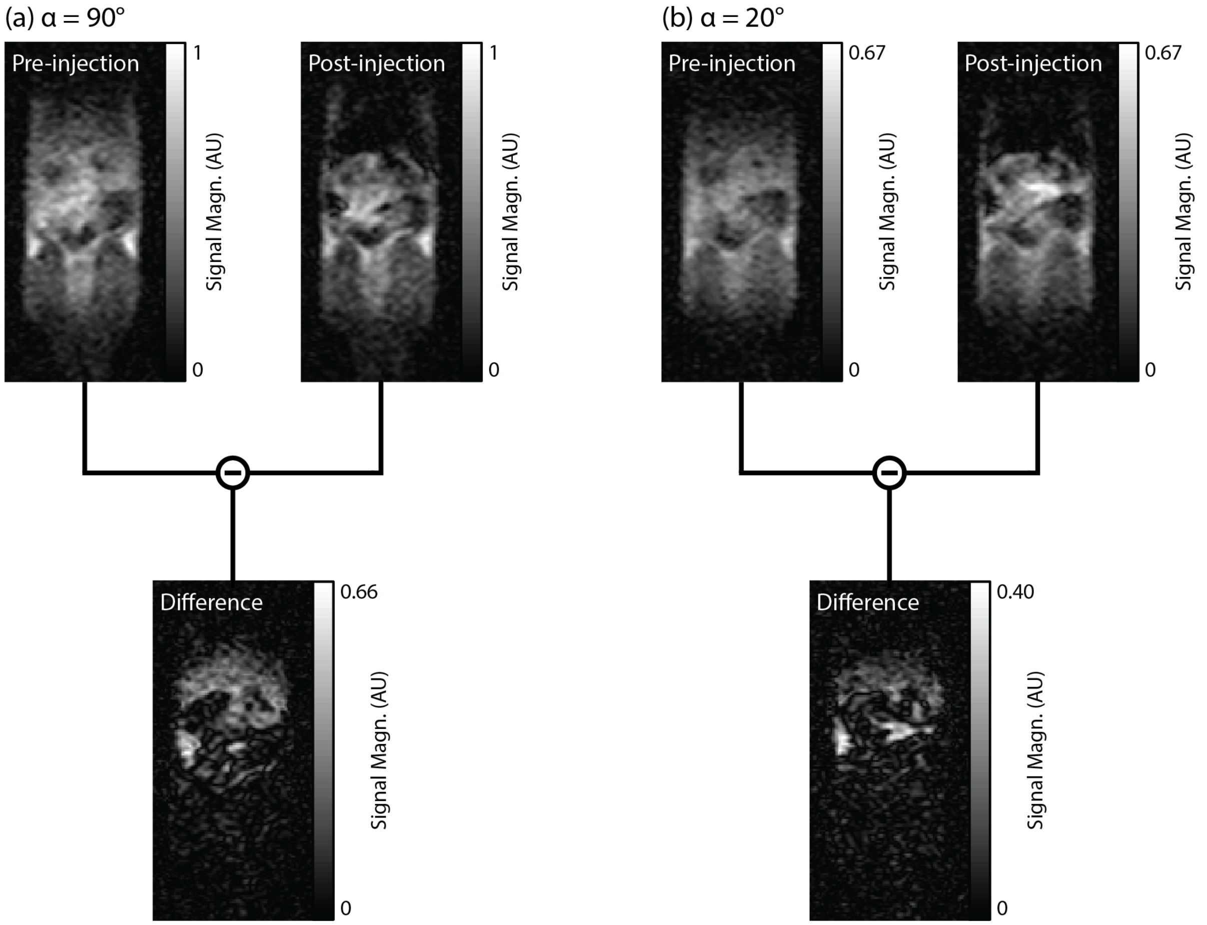

Figure S7. **Pre-injection and post-injection difference imaging highlights regions of SPION-induced MRI signal change.** **(a)** A difference image is calculated from the $\alpha = 90°$ pre-injection and post-injection slices outlined in Fig. 5 of the main text. **(b)** A difference image is calculated from the $\alpha = 20°$ pre-injection and post-injection slices outlined in Fig. 5 of the main text. MRI signal changes highlighted in the $\alpha = 90°$ difference image arise from SPION-induced shortening of T_2 which causes negative image contrast in the post-injection image. MRI signal changes highlighted in the $\alpha = 20°$ difference image arise from resonance shifts caused by SPION magnetization, which leads to positive contrast in addition to the T_2-shortening-induced negative contrast. Difference images were calculated by subtracting the signal intensity of the post-injection image slice from the signal intensity of the pre-injection image slice. Field of view in each slice is 155 mm $\times$ 73 mm. The $\alpha = 90°$ and $\alpha = 20°$ images were acquired with 6.2 min and 12.4 min scans, respectively.

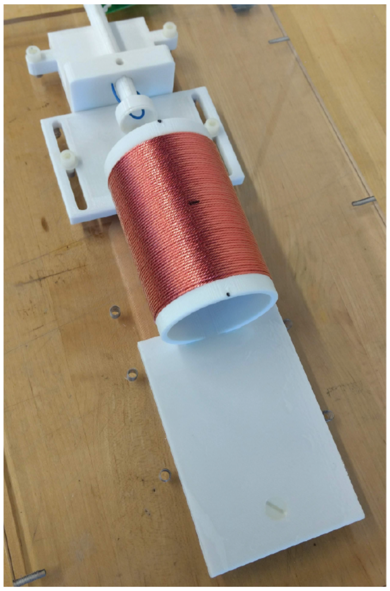

Figure S8. **Rat body coil.** The custom-built imaging coil was designed to accommodate a rat body with a high filling factor and resonated to the ^{1}H frequency of 276 kHz using an external capacitor board (series-match, parallel-tune). The nose cone at top was used for animal alignment and to deliver isoflurane anesthesia during imaging. This probe was built following the design process described in detail in Ref. 30. Photo Credit: David Waddington, The University of Sydney.
